# A magnetoelectric flux gate: new approach for weak *DC* magnetic field detection

**DOI:** 10.1038/s41598-017-09420-w

**Published:** 2017-08-17

**Authors:** Zhaoqiang Chu, Huaduo Shi, Mohammad Javad PourhosseiniAsl, Jingen Wu, Weiliang Shi, Xiangyu Gao, Xiaoting Yuan, Shuxiang Dong

**Affiliations:** 1 Department of Materials Science and Engineering, College of Engineering, Peking University, Beijing, 100871 China; 2 Beijing Key Laboratory for Magnetoeletric Materials and Devices, Beijing, 100871 China

## Abstract

The *magnetic flux gate* sensors based on Faraday’s Law of Induction are widely used for *DC* or extremely low frequency magnetic field detection. Recently, as the fast development of multiferroics and magnetoelectric (ME) composite materials, a new technology based on ME coupling effect is emerging for potential devices application. Here, we report a *magnetoelectric flux gate* sensor (MEFGS) for weak *DC* magnetic field detection for the first time, which works on a similar *magnetic flux gate* principle, but based on ME coupling effect. The proposed MEFGS has a shuttle-shaped configuration made of amorphous FeBSi alloy (Metglas) serving as both magnetic and magnetostrictive cores for producing a closed-loop high-frequency magnetic flux and also a longitudinal vibration, and one pair of embedded piezoelectric PMN-PT fibers ([011]-oriented Pb(Mg,Nb)O_3_-PbTiO_3_ single crystal) serving as *ME flux gate* in a differential mode for detecting magnetic anomaly. In this way, the relative change in output signal of the MEFGS under an applied *DC* magnetic anomaly of 1 nT was greatly enhanced by a factor of 4 to 5 in comparison with the previous reports. The proposed *ME flux gate* shows a great potential for magnetic anomaly detections, such as magnetic navigation, magnetic based medical diagnosis, etc.

## Introduction

Multiferroic magnetoelctric (ME) materials have been attracting considerable interest due to the potential application, particularly in terms of magnetic field sensors, microelectromechanical system, tunable microwave devices, tunable bandpass/bandstop filters, tunable phase shifters and spintronics, etc, since the last two decades^1–6^. It has been investigated and proved that multi-phase ME composites are able to exhibit much better ME coupling effect than single phase ME materials at room temperature^7–9^. In recent years, a variety of bulk ME composites with different phase connectivity, i.e., (0-3), (1-3), (2-2), (2-1), or (1-1), have been paid extensive attention, and tremendous progress has been achieved^10–19^. To date, one-dimensional (1-1) connectivity ME composites consisting of a [011]-oriented Pb(Mg,Nb)O_3_-PbTiO_3_ (PMN-PT) single crystal fiber laminated with laser-treated amorphous FeBSi alloy (Metglas) and operating in *L-T* mode (longitudinally magnetized and transversely poled) achieved the highest resonance ME coefficient of ~7 *kV* *cm* ^−1^ *Oe* ^−1^ in the case of bulk composites^19^. In addition, ME thin film cantilever type sensors made of AlN and FeCoSiB could possess a high resonance ME coupling coefficient of 5 *kV* *cm* ^−1^ *Oe* ^−1^ in the air, and even 20 *kV* *cm* ^−1^ *Oe* ^−1^ in vacuum^20, 21^.

In spite of the significant advances, it is always and will remain an open challenge in sensing weak *DC* and extremely low frequency *AC* (ranging from 10 mHz to 10 Hz) magnetic fields due to the large 1/*f* noise^22–25^. In this respect, magnetic sensors based on *flux gate* principle, superconducting quantum interference effect (SQUIDs), tunneling magnetoresistance(TMR), anisotropic magnetoresistance (AMR), giant magnetoresistance (GMR), giant magnetoimpedance (GMI), and nonlinear magnetoelectric effect have intrigued dramatic research interests^26–30^. However, the applications of magnetic sensors are subjected to many limitations. For example, SQUIDs-based devices requires extremely low operation temperature, which leads to high manufacture and maintenance cost^26^. The need for an external magnetic bias for TMR, GMR and GMI sensors to optimize the sensitivity inevitably increases the volume and power consumption of a detection system^27–29^. In addition, the resistance variation in terms of AMR or GMR sensors is quite low in responding to weak magnetic field^27, 29, 30^. Furthermore, the frequency conversion technology based on nonlinear ME effect fails to work when it comes to the measurement of *DC* magnetic field^22, 23^. It is well known that *flux gate* sensors are able to detect small *DC* magnetic fields, typically, from 0.5 nT to 100 μT, and now they have been widely used in the market^31–36^. Unfortunately, their power consumption tends to be rather high since the requirement to saturate the magnetic core materials periodically^37^.

In this paper, we report a shuttle-shaped, non-biased *magnetoelectric flux gate* sensor (MEFGS) enlightened by the working mechanism and configuration of the *magnetic flux gate* sensors^31, 35^. We have studied its working mechanism in comparison with *flux gate* sensors and characterized the ME performance as well. In addition, we also investigate its absolute *DC* magnetic field sensitivity in comparison with those based on the conventional magnetic detection technologies. We will see the relative change in output signal of the MEFGS under an applied *DC* magnetic anomaly of 1 nT was greatly enhanced by a factor of 4 to 5 in comparison with the previous reports.

## Results

### Structure design and working principle

**Figure 1:**
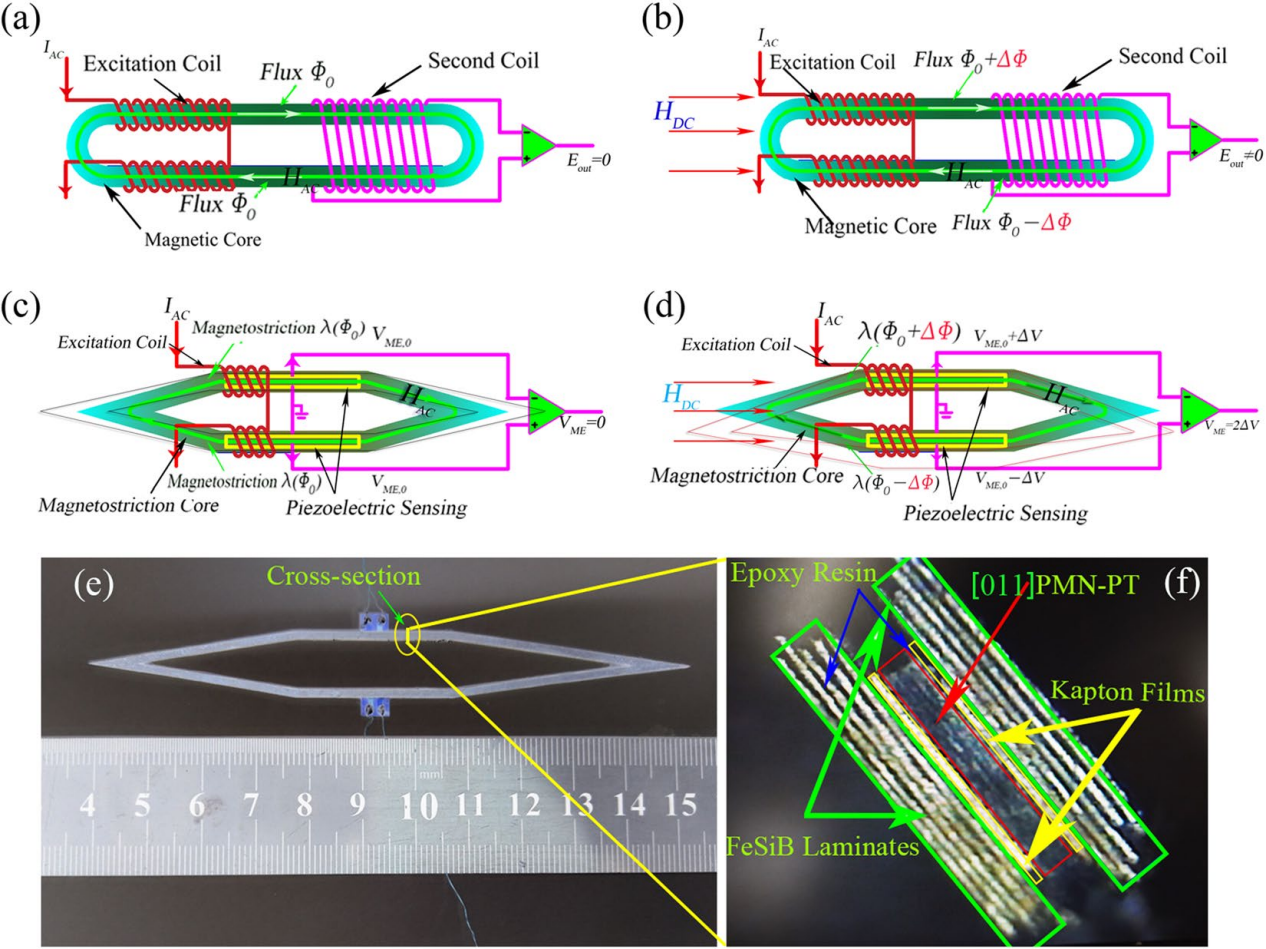
Schematic representation of the conventional flux gate senor and our proposed magnetoelectric flux gate sensor. The structure of (**a**), (**b**) a race-track *flux gate* sensor and (**c**), (**d**) our proposed *Magnetoelectric (ME) flux gate* sensor; (**a**), (**c**) in the absence of *DC* magnetic field (*H*_*DC*_); (**b**), (**d**) in the presence of *DC* magnetic field (*H*_*DC*_); (**e**) A photograph of the actual *ME flux gate*; (**f**) A cross-sectional optical image of the ME composites.

Conventional *flux gate* magnetic sensors are based on Faraday’s Law of Induction for magnetic anomaly detection. Figure 1(a) and (b) show the structure and working principle of a racetrack *magnetic flux gate* sensor. As shown in Fig. 1(a), the *flux gate* sensor is composed of a racetrack type magnetic core surrounded by an excitation (first) coil and a detection (second) coil. The first coil is used for exciting an *AC* magnetic field *H*_*AC*_, which produces a closed-loop flux Ф_0_ in the racetrack magnetic core and saturates the magnetic core materials periodically. The second coil is used for detecting magnetic anomaly (*H*_*DC*_)^35^. In the case of (*H*_*DC*_) = 0, the output of the *magnetic flux gate* sensor could be absent in theory, since the net magnetic flux Ф (=Ф_0_ − Ф_0_) passing through the second coil is zero, as shown in Fig. 1(a). However, we could expect an effective output signal E_out_ =  − 2dΔФ/dt, from the second coil in the presence of *DC* magnetic field *H*_*DC*_, because there is a net magnetic flux Ф ( = 2ΔФ) passing through the second coil, see Fig. 1(b).

In a similar way as described in Fig. 1(a) and (b), a *magnetoelectric flux gate* sensor (MEFGS) is designed into a shuttle-shaped structure (instead of a racetrack structure) with an excitation coil and one pair of piezoelectric sensing elements (instead of second coil), as shown in Fig. 1(c) and (d). When a constant *AC* current *I*_*AC*_ passes through the excitation coil, a closed-loop high-frequency magnetic field *H*_*AC*_ and flux Ф_0_ can be excited in the magnetic core, which induces a symmetric elongating and shrinking of two halves due to the magnetostrictive effect. Therefore, a longitudinal vibration mode of the shuttle-shaped structure is produced, and correspondingly, the difference output ME signal from ME gate will be zero due to symmetric vibration in the absence of an applied *DC* magnetic field, as shown in Fig. 1(c) with dash line.

However, once a *DC* magnetic anomaly appears, the magnetic field increases in one half, and while it decreases in another half, which then causes unsymmetrical elongating and shrinking in two halves of the structure. As a result, the initial longitudinal vibration mode of the shuttle-shaped structure tends to be a longitudinal-bending one due to the effect of magnetic anomaly *H*_*DC*_, as shown in Fig. 1(d) with dash line. In this case, the differential signal output from *ME flux gate* will be non-zero.

**Figure 2:**
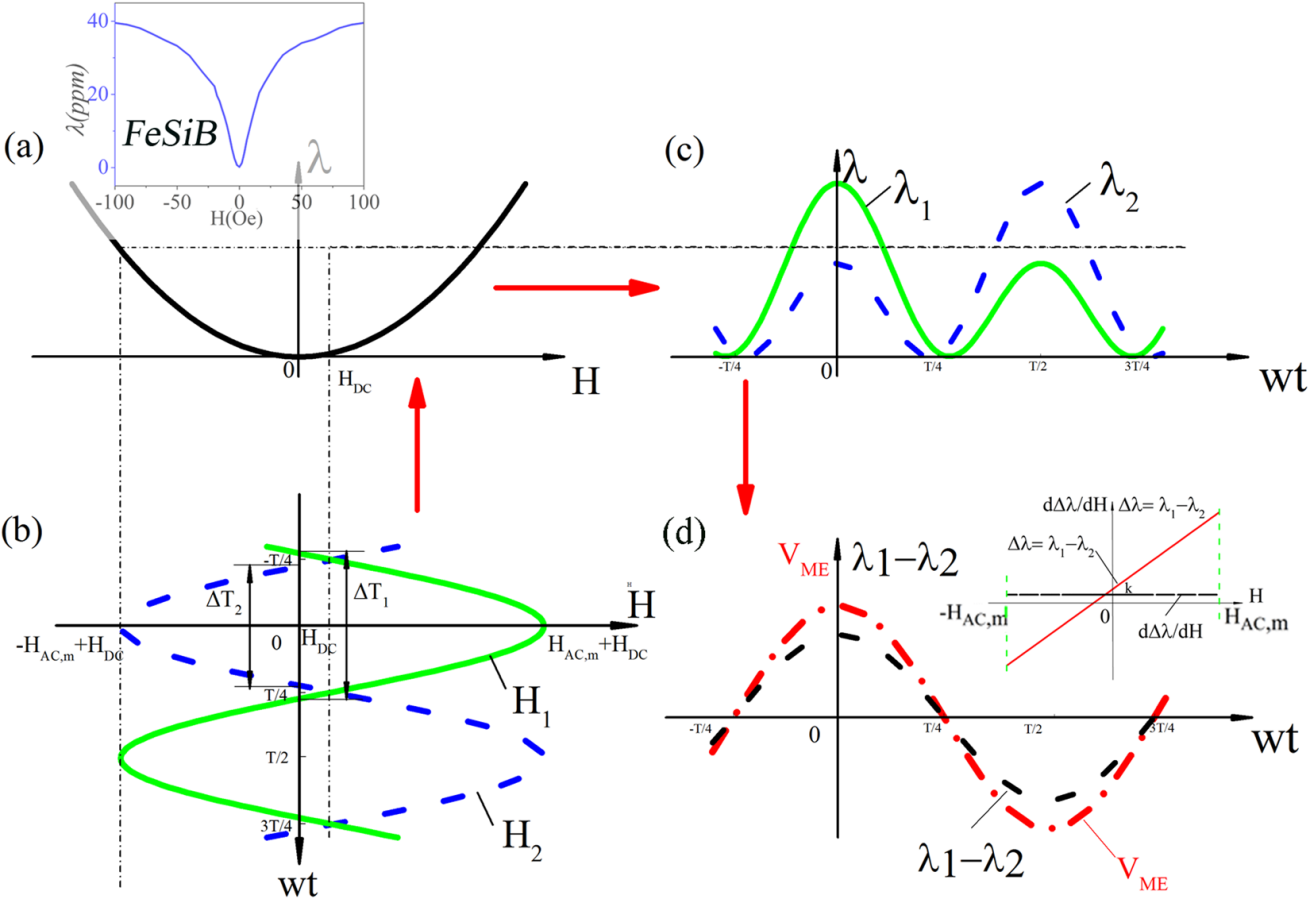
The working principle of our proposed magnetoelectric flux gate sensor. (**a**) The magnetostrictive curve of magnetic core; (**b**) the magnetic field in top half: (*H*_*AC*_ + *H*_*DC*_), while in bottom half: (−*H*_*AC*_ + *H*_*DC*_); (**c**) The double-frequency magnetostrictions of *λ*_1_ and *λ*_2_ in time domain for two half magnetic cores (1# and 2#); (**d**) The magnetostrictive difference Δ*λ* and the ME signal difference $\Delta {V}_{ME}$ of two halves in time domain in same-frequency first harmonic signal form; the inset is Δ*λ* as a function of *H*_*DC*_ and its derivative with respect to *H*_*DC*_.

With respect to a *flux gate* sensor, the induced *E*_*out*_ from the second coil under *H*_*DC*_ can be found as ref. 34

1 $${E}_{out}=-{N}_{s}{A}_{s}\frac{d(\Delta B)}{dt}=-2{N}_{s}{A}_{s}\frac{{d}^{2}B}{dHdt}{H}_{DC},$$

in which *E*_*out*_ is proportional to *H*_*DC*_. Note that *E*_*out*_ is a *second harmonic signal* relative to *H*_*AC*_. When it comes to the proposed *ME flux gate* sensor, the working mechanism, which is similar to that of *flux gate* sensor, is illustrated in Fig. 2. The magnetostrictive curve is assumed to be a quadratic function of *H* (*λ* = *kH* ^2^, where *k* is a parameter related to the magnetostrictive performance of magnetic material), as shown in Fig. 2(a). Here assuming the exciting magnetic field *H*_*AC*_ = *H*_*m*_cos(ωt) is small. In the case without an external magnetic field *H*_*DC*_, *H*_*AC*_ in top half of the shuttle-shaped magnetostrictive core is equal to that in bottom half in amplitude, but they are in reverse phase. *H*_*AC*_ then excites two symmetric but frequency-doubling magnetostrictions *λ*_1_(*H*_AC_) and *λ*_2_(−*H*_AC_) in two halves of the shuttle-shape magnetic core. Therefore, the induced two ME signals from one pair of piezoelectric sensing elements will be completely identical, resulting a zero output signal from *ME flux gate* due to its differential mode. However, when an additional external magnetic field *H*_*DC*_ appears, see Fig. 1(d), the magnetic field in top half will become (*H*_*AC*_ + *H*_*DC*_), while in bottom half it becomes (−*H*_*AC*_ + *H*_*DC*_), as they are shown in a time domain in Fig. 2(b). Therefore, the magnetostrictions *λ*_1_(*H*_*AC*_ + *H*_*DC*_) produced in top half and *λ*_2_(−*H*_*AC*_ + *H*_*DC*_) in bottom half will become asymmetric, as they are presented in time domain in Fig. 2(c). The asymmetric magnetostrictions in two halves further induce asymmetric two ME signals due to ME coupling effect, and their differential output ΔV_ME_ from *ME flux gate* is non-zero that is directly proportional to the external magnetic field *H*_*DC*_, see Fig. 2(d).

Now we try to find the relationship between ΔV_ME_ and *H*_*DC*_. Assuming a *DC* magnetic filed *H*_*DC*_ appearing along longitudinal axis direction, as shown in Fig. 1(d), and further assuming the applied *H*_*DC*_ is small, the magnetostrictive difference ${\rm{\Delta }}{\rm{\lambda }}$ between two halves is

2 $$\Delta \lambda ={\lambda }_{1}-{\lambda }_{2}\approx 2\frac{d\lambda }{dH}{H}_{DC}.$$

It is well known the output ME voltage *V*_*ME*_ can be written as ref. 23

3 $${V}_{ME}={\alpha }_{ME}\cdot {t}_{p}\cdot {H}_{AC}=\frac{\partial E}{\partial \sigma }\cdot \frac{\partial \sigma }{\partial \lambda }\cdot \frac{\partial \lambda }{\partial H}\cdot {t}_{p}\cdot {H}_{AC},$$

where *α*_*ME*_ is the ME coupling coefficient, σ is the mechanical stress, and *t*_*p*_ is the thickness of the piezoelectric phase. Considering that the *ME flux gate* sensor working in a differential mode, the output differential ME voltage $\Delta {V}_{ME}$ can be expressed as follow^23^:

4 $${\rm{\Delta }}{V}_{ME}={t}_{p}\frac{\partial E}{\partial \sigma }\frac{\partial \sigma }{\partial \lambda }\frac{\partial ({\rm{\Delta }}\lambda )}{\partial H}{H}_{AC}=-{t}_{p}\frac{{g}_{32,p}}{{s}_{33}^{\ast }}2k{H}_{AC}{H}_{DC},$$

where ${g}_{32,p}=-\frac{\partial {E}_{3}}{\partial {\sigma }_{2}}\,\,$is the transverse piezoelectric voltage constant of the [011]-oriented PMN-PT;$\,{s}_{33}^{\ast }=1/(\frac{\partial {\sigma }_{3}}{\partial {\lambda }_{3}})$ is the equivalent elastic compliance of the ME composite; $k=\frac{{\partial }^{2}\lambda }{\partial {H}^{2}}$ is a constant associated with material properties. The output differential ME voltage Δ*V*_*ME*_ in time domain is *first harmonic signal*, as shown in Fig. 2(d). Note that the ME voltage Δ*V*_*ME*_ is proportional to both *H*_*DC*_ and *H*_*AC*_.

It should be pointed that if the *DC* magnetic field is completely absent, the magnetostrictions of the two halves of the shuttle-shape magnetic core exhibit *frequency-doubling effect*. Frequency doubling and harmonic distortion behaviors in laminated ME composite have been previously studied theoretically and experimentally^38–42^. *Dmitrii et al*. reported a *DC* magnetic field detection with a responsibility of 2.5 V/mT based on the nonlinear magnetoelectric effect of a well-known sandwiched ME structure^42^. In the shuttle-shaped *ME flux gate* sensor, from the Equation 4, the output voltage ${\rm{\Delta }}{V}_{ME}$ should be zero in theory when there is no applied field *H*_*DC*_. Once a *H*_*DC*_ appears, ${\rm{\Delta }}{V}_{ME}$, which is proportional to *H*_*DC*_, is induced, and it exhibits the same-frequency harmonic signal as that of *H*_*AC*_, i.e., the frequency-doubling phenomenon will disappear. Clearly, “frequency-doubling” to “same-frequency” phenomenon may be an important criterion to indicate *H*_*DC*_ appearing. While differing from it, the “same-frequency” to “*frequency-doubling*” phenomenon may be an important criterion to indicate *H*_*DC*_ appearing for a *flux gate* sensor. Therefore, the proposed *ME flux gate* sensor offers a new approach for *DC* magnetic field detection, and it will be expected to possess the equal or even better performance for sensing extremely weak *DC* magnetic field in comparison with *magnetic flux gate* sensor, but it has much lower power consumption and also much smaller sizes.

### Materials fabrication and mechanism confirmation

**Figure 3:**
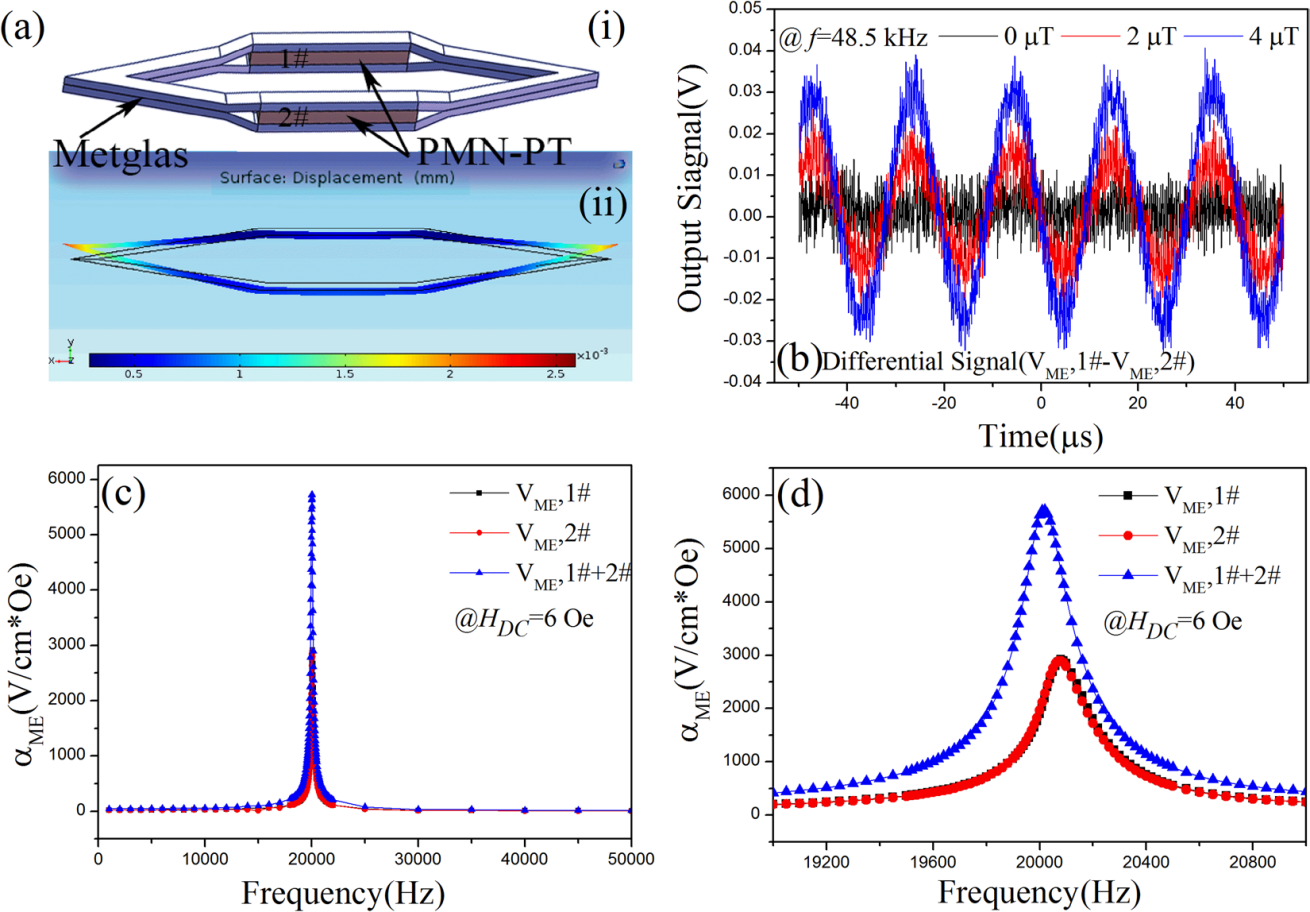
The experimental verification concerning the working principle and the ME performance for our proposed ME Gate sensor. **(a-i**) Three-dimensional view of the ME flu gate sensor made of a laser-treated Metglas magnetic core (shuttle shape) and one pair of [011]-oriented PMN-PT single crystal fibers (as labeled as 1# and 2#) embedded in the magnetic core; (**a-ii**) A schematic view of the forced vibration mode at non-resonant frequency. (**b**) The differential signal output of the MEFGS in response to varying DC magnetic field at 0 μT, 2 μT, 4 μT, respectively; The frequency response of ME coupling coefficient ${\alpha }_{ME,1\#}$, ${\alpha }_{ME,2\#}$ and ${\alpha }_{ME,1\#+2\#}$ of the MEFGS in frequency range from 1 to 50 kHz (**c**), and 19 to 21 kHz (**d**). Here,$\,{\alpha }_{ME,1\#+2\#}$ is the sum ME signal output of two halves of the MEFGS when the two terminals are connected in series.

We fabricated a *magnetoelectric flux gate* sensor. The magnetic core of MEFGS is made of laser-treated amorphous FeBSi alloy (Metglas) which has a high permeability and also piezomagnetic effect. The methods concerning the laser treatment and the sensor fabrication have been reported by *Chu et al*.^19^. One pair of [011]-oriented PMN-PT single crystal fibers (as labeled as 1# and 2#) were embedded in the magnetic core. Figure 1(e) shows the prototype of our proposed *ME flux gate* sensor and the cross-sectional optical microscopy image of the ME composite is shown in Fig. 1(f). The three-dimensional view of the *ME flux gate* sensor and its forced vibration mode (at non-resonant frequency) under *H*_*AC*_ excitation are presented in Fig. 3(a-i) and (a-ii), respectively. We then verified the theoretical analysis concerning the proposed MEFGS experimentally. Figure 3(b) shows differential signal output ${\rm{\Delta }}{V}_{ME}$ (at non-resonant frequency *f* = 48.5 kHz) under different magnetic anomaly *H*_*DC*_, which was observed directly from a mixed digital signal oscilloscope (Keysight 4024 A). It can be clearly seen that ${\rm{\Delta }}{V}_{ME}\,$is near zero when H_DC_ = 0 $\mu T$. Once a *DC* magnetic field of 2 or 4 μT appears, a dramatic differential ME signal (*first harmonic*) is produced from the *ME flux gate* sensor. The measured results agree well with those as expected in Fig. 2(d).

Figure 3(c) shows the measured frequency responses of ME electric field coefficient ${\alpha }_{ME}$ of the MEFGS. The ME coupling coefficient ${{\rm{\alpha }}}_{{\rm{ME}}}$ for each half of the shuttle shape ME sensor are 22.6 and 22.2 $\,V/{\rm{cmOe}}$ at non-resonance; while at resonance frequency of 20.02 kHz, it dramatically increases to 2918 and 2894 $\,V/\mathrm{cm}\ast \mathrm{Oe}$, respectively. It can be seen from the measured frequency spectrum characteristics as presented in Fig. 3(d) that the two halves of the MEFGS structure exhibit quite similar ME performance, proving that they are wonderful symmetric. The sum output of the two halves even reaches to 5700 V/cmOe, which is close to the best result reported in one dimensional (1-1) ME composite^19^.

It is widely known that ME composite materials exhibit the strong magnetoelectric coupling at resonance, therefore, a high magnetic field sensitivity at resonant frequency should be expected. *Chu et al*. develpoed a one-dimensional [011]-oriented PMN-PT/Metgals (1-1) composite, which had a limit *AC* magnetic field sensitivity as low as 1.35 × 10^−13^ Tesla at resonant frequency^19^. *Gao et al*. reported a (2-1) Metglas/PMN–PT laminate sensors showing a *DC* magnetic field sensitivity as high as (i) 5 nT at 1 kHz and (ii) 1 nT near the resonant frequency in a shield chamber^25^.

### High sensitivity to weak DC magnetic field

**Figure 4:**
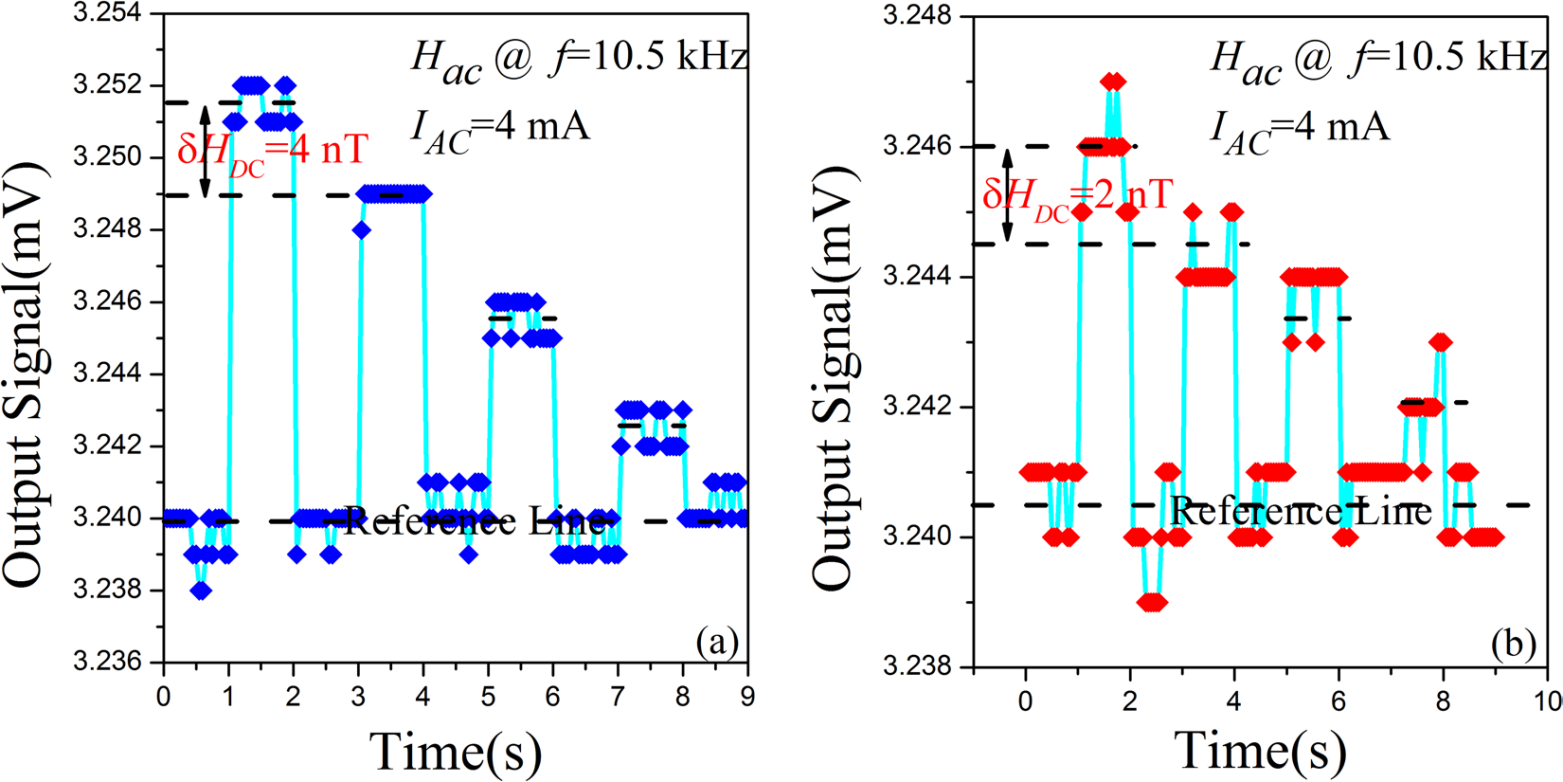
ME voltage output signal of the MEFGS in response to a weak step-varying DC magnetic field at the first-order longitudinal vibration mode (fr1 = 10.5 kHz). (**a**) In response to a step of 4 nT. (**b**) In response to a step of 2 nT.

In this work, we investigate the *DC* magnetic field responses of the MEFGS in the both cases of resonance and non-resonance frequencies. Measurements of magnetic field sensitivity was carried out in a shielded chamber with a lock-in amplifier. We found that under exciting of a closed-loop *H*_*AC*_ with the frequency of *f* = 10.5 kHz, the MEFGS exhibits a strong resonant response at *f*_*r*_ = 21 kHz, because of the frequency-double effect. We then observed *H*_*DC*_ response at *f* = 10.5 kHz due to “frequency-doubling” to “same-frequency” effect. Figure 4 shows the measured ME differential voltage response to a weak and step-varying *H*_*DC*_. As shown in Fig. 4(a), a step-varying δ*H*_*DC*_ of 4 nT could be distinguished clearly, and the ME differential signal was also able to return to the initial level. However, when further decreasing δ*H*_*DC*_ to be 2 nT, we found the noise level becomes non-ignorable, as shown in Fig. 4(b). Apparently, noise level at resonance is a main obstacle to the detection of an extremely weak *DC* magnetic field. In order to improve *DC* magentic field sensitivity, it seems that *H*_AC_ exciting at resonant frequency is unnecessary, because it could increase the undesired noise greatly (althogh it also improves ME coupling greatly). In our case, lowering noise of MEFGS and obtaining a stable vibration condition are eseential for sensing an extremely low *DC* magneitc field.

**Figure 5:**
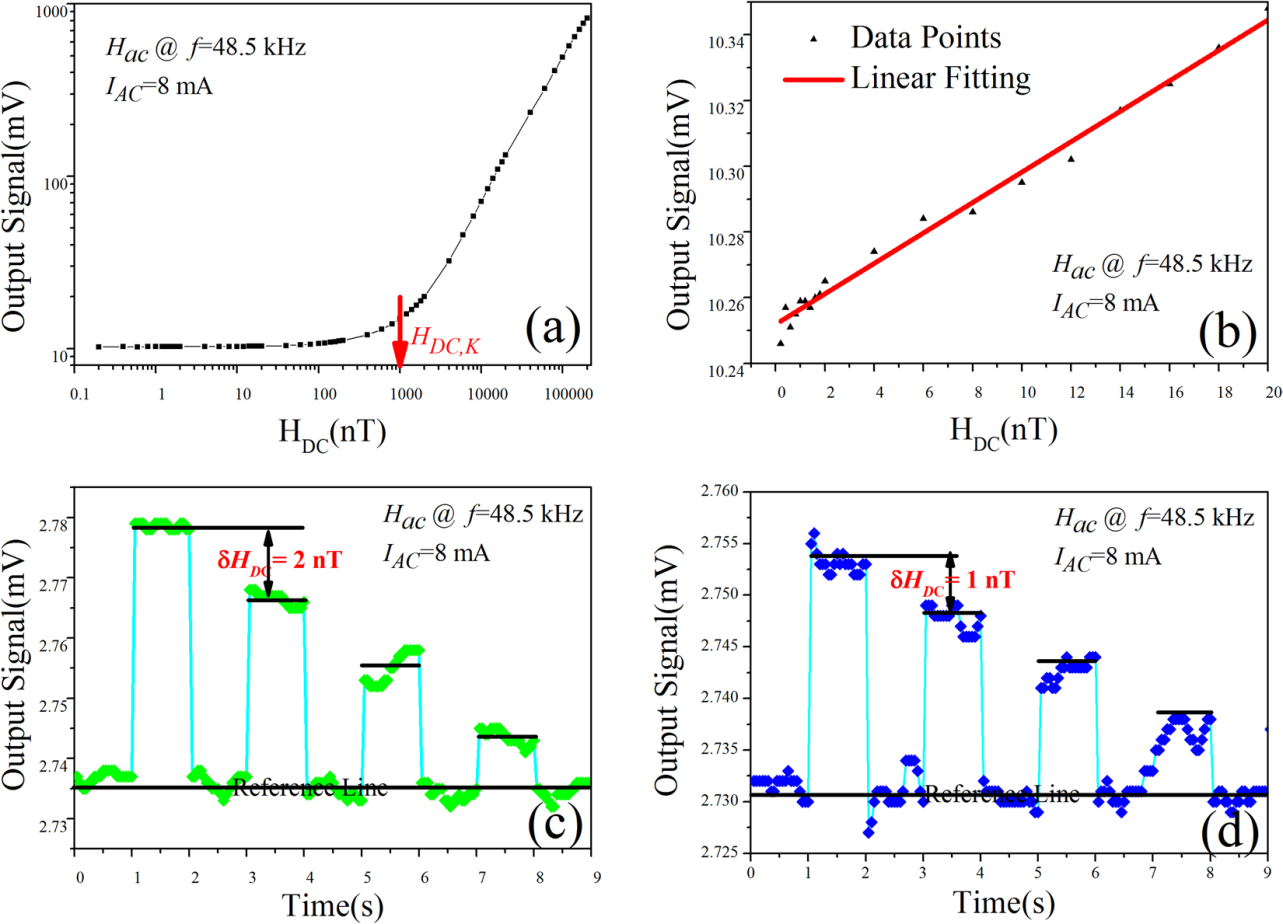
Charaterization for weak DC magneitic field measured at non-resonat frequency. (**a**) The magnetic field sensitivity in terms of DC magnetic field varying from 0.2 to $2\ast {10}^{5}$ nano Tesla at non-resonant frequency of 48.5 kHz. (**b**) The zoomed area of (**a**) with DC magnetic field starting from 0.2 to 20 nano Tesla. ME voltage output signal in response to an extremely weak step-varying DC magnetic field of 2 nT (**c**)and 1 nT (**d**) at f = 48.5 kHz, which is far deviate from the resonant frequency of the MEFGS.

Finally, we investigate the *DC* magnetic field responses of the MEFGS in non-resonance frequency range, including its limit of detection (LOD) of *DC* magnetic field. We chose a much higher working frequency around 48.5 kHz for exciting *AC* magnetic field in the closed-loop magnetic core of MEFGS, which is deviated far from the resonant state of the MEFGS. Since it is a non-resonance forced vibration mode, we accordingly increase the *AC* current for produing a relatively higher *H*_*AC*_. Figure 5(a) shows the measured differential ME voltage output signal in response to an applied *H*_*DC*_ varying from 0.2 to 200000 nT. Apparently, there is a *knee H*_*DC,K*_ or threshold about 1000 nT. When *H*_*DC*_ is higher than *H*_*DC,K*_, the MEFGS exhibits a strong linear-response to the applied *H*_*DC*_ with a large slope. While *H*_*DC*_ is lower than *H*_*DC,K*_, the MEFGS shows a weak linear-responses to *H*_*DC*_ with a small slope, which may be attributed to interface effect of ferromagnetic and ferroelectric two phase, including interface strain transfer loss and interface voltage drop at bonding layer (like a capacitor)^43^. Figure 5(b) shows the zoomed area where *DC* magnetic field is limited in a small range, starting from 0.2 to 20 nT. It is clearly seen that output signal exhibits a linear relationship with *H*_*DC*_ starting from 1 nT. Measurement values then become scattering when *H*_*DC*_ is less than 1 nT. However, the LOD value of *H*_*DC*_ can be clearly seen to be 2 nT and 1 nT, respectivelly, as presented in Fig. 5(c), and Fig. 5(d), revealing a superhigh *DC* magnetic field sensitivity of the MEFGS. It should be noted here that the relative changes of the ME voltage output signal in response to 1 nT is only 0.2%, but it is still nearly 4 to 5 times higher in comparison with previously measurements on 1 nT *DC* magnetic field detection^25^. This result is significant, which shows that a *ME flux gate* sensor under exciting of non-resonant high frequency *H*_AC_ may have higher *DC* magnetic field sensitivity due to lower vibration noise, which is apparently different from the previous claims in conventional ME sensors^25, 44^. In addition, there is no need to apply a bias field for achieving a LOD value of 1 nT in present work.

## Discussion

To further improve the MEFGS, following jobs are necessary: (i) improving symmertry about two halves of the MEFGS structure for lowering noise; (ii) decreasing interface effect of ferromagnetic and ferroelectric two phases for lowering *knee H*_*DC,K*_; and (iii) optimizing *ME* working mode for obtaining a more stable performance. (iv) seeking piezoelectric materials with lower dielectric loss and piezomagetic materials with higher piezomagetic property. Proposed MFFGS shows high *DC* field detection sentivity that has significant potential toward pratical application in magnetic navigation systems and medical diagnosis.

In summary, a *DC* magnetic field sensor working on magnetoelectric coupling effect and *flux gate* principle (named as *MEFGS*) is proposed for the first time; its principle in comparison with the conventional *flux gate* sensor is analyzed theoretically and then confirmed experimentally. MEFGS is composed of a shuttle-shaped, laser-treated, and multilayered amorphous FeBSi alloy (Metglas) magnetic core and one pair of embedded [011]-oriented Pb(Mg,Nb)O_3_-PbTiO_3_ (PMN-PT) single crystal fibers. The fabricated MEFGS exhibits the strong ME coupling: non-resonance ME coefficients of two halves in the shuttle-shape structure are 22.6 and 22.28 V/cm Oe, respectively; while at resonance, they are dramatically increased to 2918 and 2894 $\,V/\mathrm{cm}\,\mathrm{Oe}$, respectively. These results also show well symmetry of the shuttle shape structure, which is important to a flux gate magnetic sensor due to its differential working mode. It was further found that the MEFGS has a wide response to *DC* magnetic field ranging from 0.2 to 200000 nT, accompanying a *knee H*_*DC,K*_ or threshold phenomenon at *H*_*DC*_ = 1000 nT possibally due to interface effect. The repeatable, absolute limit of detection (LOD) of *DC* magnetic field is found to be 1 nT at non-resonant frequency and at room temperature without bias magnetic field. The relative change of output signal of the MEFGS at 1 nT is 4 to 5 times higher in comparison with the previous reports. Proposed MEFGS can be viewed as a potential alternative ultra-sensitive *DC* magnetic field sensor, like *flux gate* sensor in particular, which shows significant availability toward practical application in magnetic navigations and magnetic based medical diagnosis because of very low power consumption and fine structure.

## Methods

### ME performance characterization

the ME coefficient was measured at room temperature using a homemade setup. A purchased Helmholtz coil and a *DC* current supply (IT6932A, ITECH, USA) were used to produce a direct current (*DC*) magnetic field bias (0–6 Oe). And a home-made Helmholtz coil carrying a standard *AC* current (6221, Keithley, USA) was used to generated an *AC* magnetic field. The induced voltages from two piezoelectric sensing elements (PMN-PT single crystal,1# and 2#) were measured with a lock-in amplifier (SR850, Stanford Research, USA), the time constant (integration time) and the bandwidth of which were set at 100ms and 1.2 Hz, respectively.

### DC magnetic field measurement

As presented in Fig. 1(d), the two halves of the magnetic core are exposed to a common applied *DC* filed *H*_*DC*_ (a small quantity) and to the *AC* excitation field *H*_1_ and *H*_2_, respectively. Since the two terminals were winded with an opposite direction, *H*_1_ is completely reverse to *H*_2_. Here the *DC* magnetic field was excited with a purchased solenoid coil carrying a standard *DC* current (6221, Keithley, USA) and the AC magnetic field was generated through home-made coils (the number of turns is 200 for each half as shown in Fig. 1(d)) excited by a Function/Arbitrary Waveform Generator (Agilent 33522 A). In addition, the induced differential voltage variations from two piezoelectric sensing elements (PMN-PT single crystal, 1# and 2#) were measured with a lock-in amplifier (SR850, Stanford Research, USA).

### Data availability

The datasets generated analysed during the current study are available from the corresponding author on reasonable request.
